# Genomics-based insights into the expanded diversity and adaptation strategies of hadal trench anammox bacteria

**DOI:** 10.1093/ismeco/ycag011

**Published:** 2026-03-13

**Authors:** Yao Xiao, Rui Zhao, Weishu Zhao, Pudi Wang, Xiang Xiao, Xiaotong Peng, Hongmei Jing

**Affiliations:** State Key Laboratory of Deep-Sea Science and Intelligent Technology, Institute of Deep-sea Science and Engineering, Chinese Academy of Sciences, Sanya 572000, China; HKUST-CAS Sanya Joint Laboratory of Marine Science Research, Chinese Academy of Sciences, Sanya 572000, China; University of Chinese Academy of Sciences, Beijing 100049, China; State Key Laboratory of Deep-Sea Science and Intelligent Technology, Institute of Deep-sea Science and Engineering, Chinese Academy of Sciences, Sanya 572000, China; State Key Laboratory of Microbial Metabolism, School of Life Sciences and Biotechnology, Shanghai Jiao Tong University, Shanghai 200240, China; State Key Laboratory of Deep-Sea Science and Intelligent Technology, Institute of Deep-sea Science and Engineering, Chinese Academy of Sciences, Sanya 572000, China; HKUST-CAS Sanya Joint Laboratory of Marine Science Research, Chinese Academy of Sciences, Sanya 572000, China; University of Chinese Academy of Sciences, Beijing 100049, China; State Key Laboratory of Microbial Metabolism, School of Life Sciences and Biotechnology, Shanghai Jiao Tong University, Shanghai 200240, China; State Key Laboratory of Deep-Sea Science and Intelligent Technology, Institute of Deep-sea Science and Engineering, Chinese Academy of Sciences, Sanya 572000, China; State Key Laboratory of Deep-Sea Science and Intelligent Technology, Institute of Deep-sea Science and Engineering, Chinese Academy of Sciences, Sanya 572000, China; HKUST-CAS Sanya Joint Laboratory of Marine Science Research, Chinese Academy of Sciences, Sanya 572000, China

**Keywords:** anammox bacteria, hadal trenches, microbial ecology, evolution

## Abstract

Anaerobic ammonium oxidation (anammox) bacteria are an important functional guild in the nitrogen cycle and contribute up to 50% of nitrogen loss in the global ocean. Hadal trenches have been recognized as a hotspot of marine biogeochemical cycles; however, the metabolic traits, ecological adaptations, and potential origins of anammox bacteria in this critical habitat remain largely unexplored. Here, we reconstructed eight anammox metagenome-assembled genomes from sediments of four hadal trenches (Diamantina, Kermadec, Mariana, and Yap), which represent four out of the five distinct anammox bacterial families (i.e. *Candidatus* Scalinduaceae, *Ca.* Anammoxibacteraceae, *Ca.* Subterrananammoxibiaceae, and *Ca.* Bathyanammoxibiaceae). The dominant trench anammox bacteria, affiliated with *Ca.* Scalindua, were similar to those found in shallow coastal sediments and oxygen-deficient seawaters. Beyond the core anammox metabolism, the hadal *Ca.* Scalindua genomes contain genes encoding cyanase and urease, indicating that they can utilize cyanate and urea besides ammonium to thrive in the hadal trenches. Compared to trench-derived *Ca.* Subterrananammoxibiaceae and *Ca.* Bathyanammoxibiaceae, ABC-type Fe^3+^ transporter and sulfate transporter *Cys*Z could help trench-derived *Ca.* Anammoxibacteraceae genomes to uptake Fe^3+^ and synthesize sulfur-containing amino acids. Molecular clock analysis suggests that the ancestors of the hadal anammox bacterial lineages appeared on Earth 1.46–0.07 billion years ago, significantly earlier than the geological formation of the trenches. The first hadal anammox bacteria were likely derived from shallower sediments and were transported into the trenches via sediment wasting. Overall, our study reveals a remarkable diversity of hadal anammox bacteria and their origin as well as survival strategies in hadal sediments.

## Introduction

Hadal trenches are an extreme oceanic habitat characterized by extremely high hydrostatic pressure (>60 MPa), permanent darkness, and low temperature (<4°C) [1]. Despite the extreme physical conditions, hadal trenches are considered hotspots of elemental cycling due to the elevated organic matter deposition and active microbial communities [2–4], which presumably promote anaerobic microbial processes [5]. Indeed, through experimental observations and modeling approaches, previous studies have revealed that anaerobic processes like denitrification and anammox can continuously remove bioavailable nitrogen species, including ammonium (NH_4_^+^), nitrite (NO_2_^−^), and nitrate (NO_3_^−^), from sediments of the Izu–Ogasawara, Mariana, Atacama, and Kermadec Trenches [6–8]. The ongoing nitrogen loss likely leads to the elevated sediment C:N ratios and potential nitrogen limitation for benthic microbial communities [9, 10]. Despite their profound significance in the hadal trench ecosystem, microbial functional guilds responsible for different biogeochemical processes are comparatively less studied, as most microbiological studies in the hadal trench have focused on the overall microbial communities [11–15] with only a few targeting specific functional groups [16–18].

Anaerobic ammonium oxidation (anammox) is among the critical functional guilds in hadal trench sediments. Thamdrup *et al*. [7] have revealed that anammox is the dominant nitrogen removal pathway in sediments of the Atacama and Kermadec Trenches. Through 16S ribosomal RNA (rRNA) gene amplicon analysis, they also suggested that hadal anammox bacterial communities are dominated by members of the genus *Candidatus* Scalindua and are highly related to those found in oxygen-deficient seawater [7]. Similarly, by using the anammox-specific functional genes (*hzsA* and *hao*), Nunoura *et al*. [6] suggested *Ca.* Scalindua is the dominant anammox bacterial lineage in sediments of the Izu–Ogasawara Trench. Although genome reconstruction is a powerful tool to uncover more information about microbes in natural environments [19], only a handful of genomes of anammox bacteria have been reported in this habitat [20–23]. In particular, Zhou *et al*. [21] recovered only two *Ca.* Scalindua genomes from Mariana Trench sediments, while Huang *et al*. [23] reported metagenome-assembled genomes (MAGs) affiliated with *Ca.* Scalinduaceae and *Ca.* Bathyanammoxibiaceae [24] in the Mariana Trench. Given that the phylogenetic width of anammox bacteria has been expanded recently, especially by the family-level additions of *Ca.* Bathyanammoxibiaceae [24], *Ca.* Anammoxibacteraceae [25], and *Ca.* Subterrananammoxibiaceae [26], it is unclear whether the above studies delivered an accurate characterization of hadal anammox communities. More importantly, extensive genome reconstruction with precise classification information is also crucial to deciphering the adaptation mechanisms and evolutionary trajectories of anammox bacteria in the hadal environments.

In an effort to fully understand anammox bacteria in the hadal realm, we leverage the metagenome sequencing datasets of four hadal trenches (Diamantina, Kermadec, Mariana, and Yap) to recover anammox bacterial genomes. We characterized their diversity at the genome level to understand the distribution of anammox bacteria and to investigate the adaptive and evolutionary processes involved. Our study provides critical insights into the mechanisms underlying nitrogen cycling in one of the least explored yet biogeochemically active regions of the global ocean.

## Materials and methods

### Sample collection

**Figure 1:**
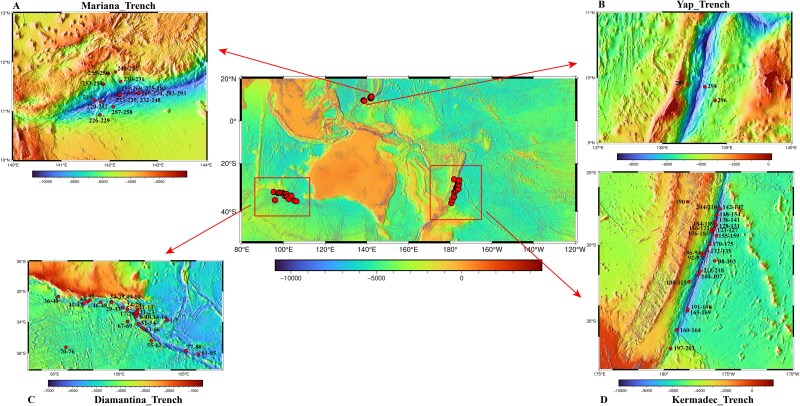
Sampling sites of the four hadal trenches investigated in this study. (A) Mariana Trench (75 samples), (B) Yap Trench (3 samples), (C) Diamantina Trench (85 samples), and (D) Kermadec Trench (133 samples). Sample information is given in Supplementary Table S3.

Four trench sites were included in this study. Kermadec and Diamantina Trenches are both located in the southern hemisphere near Australia. The Kermadec Trench reaches a maximum depth of 10 047 m and is located ~120 km off the north-eastern coast of New Zealand [27]. The Diamantina Trench is around 1500 km west of Perth, Australia, in the Indian Ocean and has a maximum depth of ~8047 m [28]. The Mariana and Yap Trenches are both located in the western Pacific Ocean of the northern hemisphere, and the southern Mariana Trench intersects the north–south trending Yap Trench [29]. Sediment samples were collected using a push core from the Diamantina (max. depth of 7003 m) and Kermadec (max. depth of 10 100 m) Trenches during cruise TS29 from November 2022 to March 2023 on R/V *Tansuoyihao* (Fig. 1, Table S3). Push cores of a maximum length of 40 cm were retrieved using the mechanical arms of the manned submersible *Fendouzhe*. Sediment cores for microbiological analyses were sliced into 2 cm-thick layers before immediately frozen at −80°C on board for subsequent onshore laboratory DNA extraction. The in situ hydrographical parameters (i.e. depth and location) were recorded by the “human”-occupied vehicles during sampling.

Besides Diamantina and Kermadec Trenches, we also investigated anammox bacteria in sediments of the Mariana Trench (max. depth of 10 911 m) and the Yap Trench (max. depth of 6578 m), by using the public data of the Mariana [22] and Yap [12] Trenches (Table S3). We also checked the distribution of putative anammox bacteria in the Atacama Trench sediment cores by using the 16S rRNA gene amplicon sequencing data of Schauberger *et al*. [30]. The data analysis procedure was described in Zhao *et al*. [31].

### DNA extraction

Total sediment DNAs were extracted from sediment samples using the DNeasy PowerSoil Pro kit (QIAGEN, USA) according to the manufacturer’s instructions. DNA concentration was measured using the Qubit dsDNA Assay kit in combination with a Qubit 2.0 Fluorometer (Life Technologies, USA) and verified by 1% agarose gel electrophoresis. Sequencing libraries were generated using the NEBNext Ultra DNA Library Prep kit for Illumina (NEB, USA) and sequenced using a NovaSeq 6000 platform (Illumina, USA). Clean data (150 bp paired-end reads) were obtained by removing the adapters and barcodes, and reads containing poly-N or that were of low quality from the raw data using the FASTX-Toolkit (http://hannonlab.cshl.edu/fastx_toolkit) and were subsequently checked by FastQC (https://github.com/s-andrews/FastQC).

### Metagenomic sequencing, assembly, and binning

High-quality short reads of each sample were assembled using MEGAHIT v1.2.9 with parameters “--k-min 21 --k-max 141 --k-step 10.” Genomic binning was implemented using MetaWRAP v1.3 [32] binning module based on maxbin2, metabat2, and concoct methods, with 1.5 kb as the contig length cut-offs. The MAGs were refined using the “bin_refinement” module of MetaWRAP v1.3 [32]. All MAGs were dereplicated for species-level clustering using dRep v3.5.0 [33] with an average nucleotide identity (ANI) cutoff value of 95%. The quality of MAGs was checked using the “predict” command of CheckM2 v1.1.0 [34] with default settings, and only those MAGs meeting the high-quality threshold of the minimum information about a metagenome-assembled genome (MIMAG) standards (completeness ≥75% and contamination ≤10%) were retained for further analysis. The estimated genome size was calculated based on the total MAG size and completeness. The taxonomic classifications of representative MAGs were annotated using GTDB-TK v2.4.0 with reference to the GTDB R220 database [35].

### Relative abundance calculation and function annotation for trench anammox bacteria

To determine the distribution of anammox bacteria in different trenches, the existing trench metagenomic sequencing data were leveraged from each trench. The relative abundance of each anammox MAG in four families was calculated using CoverM v0.7.0 (parameters: -m relative_abundance --trim-min 0.10 --trim-max 0.90 --min-read-percent-identity 0.95 --min-read-aligned-percent 0.75) [36].

Amino acid sequences encoded by the anammox bacterial genomes were predicted using Prokka v1.14.6 [37]; protein functional annotation was performed using KofamScan v1.3.0 using the Kyoto Encyclopedia of Genes and Genomes (KEGG) database. The metabolic pathways were reconstructed using the KEGG Mapper [38]. Average amino acid identity (AAI) and ANI between genomes were calculated using EzAAI [39] and FastANI [40] with the default settings, respectively. Following Konstantinidis *et al*. [41], species were distinguished based on 65%–95% AAI and 95%–98.6% 16S rRNA gene sequence identity.

### Phylogenomic tree construction

To pinpoint the phylogenetic placements of the anammox bacteria, we performed phylogenetic analyses for them together with the high-quality genomes of the Planctomycetes phylum included in the GTDB R220 Release. The bacterial 120 single-copy genes were identified and aligned using GTDB-tk v2.4.0 and trimAl v1.4.rev15 [42] with the auto options, respectively. The maximum-likelihood (ML) phylogenetic tree was inferred based on this alignment using IQ-TREE v2.3.4 [43] with Q.yeast + F + R6 the best-fit model selected by ModelFinder [44] and 1000 ultrafast bootstrap iterations using UFBoot2 [45].

### Comparative genomic analysis

We performed a comparative analysis of four families of anammox bacteria MAGs within the trench and nontrench environments (i.e. hypoxic seawater, hydrothermal vent sediment, marine sediment, and groundwater, Table S4) using Anvi’o v8 [46] according to the pangenome analysis workflow. Representative MAGs with close phylogenetic relationship to trench-derived MAGs were selected and included in this comparison. All genomes were firstly annotated using anvi’o against the National Center for Biotechnology Information (NCBI) Clusters of Orthologous Groups (COGs) database [47]. The comparative genomic analysis using Basic Local Alignment Search Tool (BLAST) to quantify the similarity between each pair of genes and the Markov cluster algorithm [48] to resolve clusters of homologous genes. The shared and unique genes in the two genomes were identified via functional enrichment analysis [49]. Anammox-bacteria MAGs were further compared through the Anvi’o pangenome analysis pipeline [50]. A contig database for each genome was created with the following additions: Hidden Markov Model (HMM) search, single copy gene (SCG) taxonomy, transfer RNAs (tRNAs) scan, NCBI cogs search, and KEGG Kofam search. The pangenome was then visualized via the Anvi’o interactive display, organizing samples by a gene cluster frequency tree. Functional enrichment between strain categories within the pangenome analysis was computed via anvi-compute-functional-enrichment-in-pan using the COG20_PATHWAY annotation sources [49].

### Molecular dating of anammox bacteria

To estimate the divergence time of the four anammox families in trench from other environments, molecular clock analysis using the program MCMCTree from the Phylogenetic Analysis by Maximum Likelihood (PAML) package v 4.10.7 was performed [51]. The phylogenetic analysis was based on a concatenation of 26 single-copy genes selected from a set of 71 bacterial marker genes with low horizontal transfer rates [52]. The individual genes were identified in the genomes using Anvi’o v8 [46], and the individual alignment in FASTA format was converted to the PHYLIP format using Clustal Omega [53] for the subsequent MCMCTree analysis. Also based on the alignment, a maximum-likelihood phylogenetic tree was inferred using IQ-TREE v2.3.4 [43] with the best-fit Q.insect + I + R6 substitution model. The resulting phylogenetic tree was rooted at the desired branches using the tree manipulating tool nwkit [54], and the dating analysis was performed in MCMCTree with the approximate likelihood method [55]. The estimated age ranges of the following four nodes in the tree were used to calibrate the divergence time of the tree root Cyanobacteria at 3.0 Ga, node Nostocales Crown at 1.75–2.07 Ga [56], node *Aeromonas* Crown at 0.072 to 0.479 Ga [57], and node *Vibrio* Crown at 0.113–0.278 Ga [58].

## Results and discussion

### Four families of anammox bacteria found in hadal trench sediments

Through metagenome sequencing and genome reconstruction, we obtained eight dereplicated anammox MAGs from the four hadal trenches (Fig. 1). These MAGs were taxonomically classified as members of the order Brocadiales within Planctomycetota and are the focus of this study. Based on the assessment of CheckM2, the eight MAGs were classified into three high-quality (>90% completeness and <5% contamination) and five medium-quality (>50% completeness and <10% contamination), with 102–540 contigs and genome sizes of 1.8–3.7 Mbp (Table 1). All the MAGs contained at least 25 tRNA genes, 5 MAGs contained rRNA genes, and 4 MAGs harbored 16S rRNA genes (Table 1).

**Table 1:** Summary of anammox MAGs obtained from trench sediments.

Name	Quality Levels	Completeness (%)	Contamination (%)	Genome Size (Mbp)	GC Content (%)	Coding Density	Contig N50	Coding Sequences	Number of Contigs	5S rRNA	16S rRNA	23S rRNA	tRNA	Accession ID	Lineages
hadal_Sc_1	High	94.7	1.4	3.6	39.2	0.81	64 261	3536	102	1	1	1	43	JBRBQJ000000000	c__Brocadiia;o__Brocadiales;f__Scalinduaceae;g__Scalindua;s__Scalindua sp022570935
hadal_Sc_2	Medium	87.7	2.3	3.7	39.3	0.83	16 756	3832	315	0	0	0	39	JBRBQK000000000	c__Brocadiia;o__Brocadiales;f__Scalinduaceae;g__Scalindua;s__
hadal_Sc_3	Medium	75.3	6.0	2.2	39.0	0.84	16 385	2344	242	0	0	0	25	JBRBQL000000000	c__Brocadiia;o__Brocadiales;f__Scalinduaceae;g__Scalindua;s__
hadal_Ab_1	Medium	87.9	4.3	3.6	38.1	0.85	16 901	3832	336	1	1	1	35	JBRBQM000000000	c__Brocadiia;o__Brocadiales;f__Anammoxibacteraceae;g__Anammoxibacter_A;s__
hadal_Ab_2	High	94.0	2.6	3.6	38.2	0.85	14 260	3566	339	1	1	1	37	JBRBQN000000000	c__Brocadiia;o__Brocadiales;f__Anammoxibacteraceae;g__Anammoxibacter_A;s__
hadal_Ab_3	Medium	85.1	3.7	3.2	39.0	0.85	7338	3463	540	0	0	0	31	JBRBQO000000000	c__Brocadiia;o__Brocadiales;f__Anammoxibacteraceae;g__Anammoxibacter_A;s__
hadal_Ba_1	Medium	81.7	3.7	1.8	54.2	0.88	5207	2081	465	1	4	6	48	JBRBQP000000000	c__Brocadiia;o__Brocadiales;f__Bathyanammoxibiaceae;g__Bathyanammoxibius;s__Bathyanammoxibius sp030598065
hadal_Sa_1	High	90.8	2.3	2.6	42.7	0.87	11 598	2684	309	1	0	1	35	JBRBQQ000000000	c__Brocadiia;o__Brocadiales;f__JACQHT01;g__JACQHT01;s__

**Figure 2:**
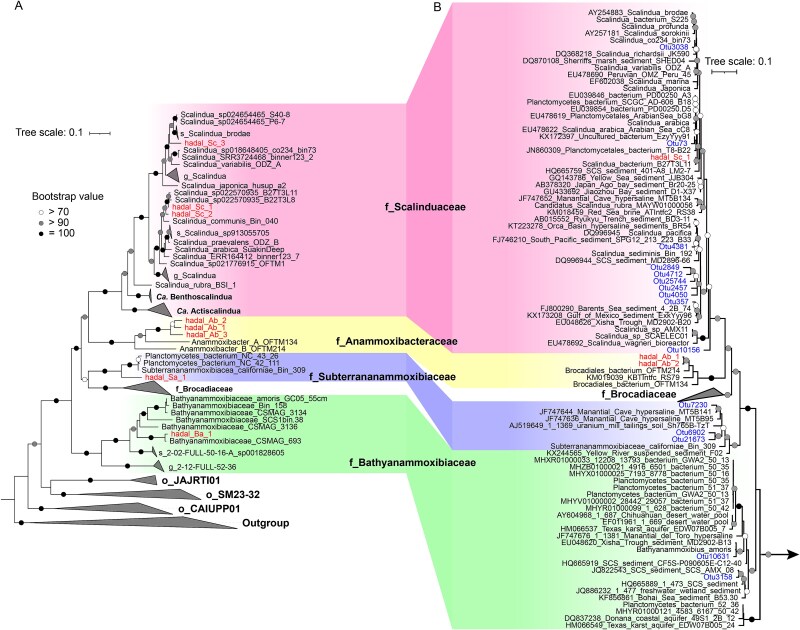
Phylogeny of the hadal trench anammox bacteria. (A) Maximum-likelihood phylogenetic tree constructed with the concatenated 120 universal bacterial marker genes. (B) Maximum-likelihood phylogenetic tree of anammox bacteria based on the 16S rRNA gene. Anammox bacterial families in both trees are marked and connected by bands of the same color. MAGs recovered from hadal sediments in this study are highlighted. Bootstrap values of >70 (*n* = 1000) were shown with symbols listed in the legend. The scale bars show estimated sequence substitutions per residue.

The identities and phylogenetic affiliations of the anammox bacterial genomes were analyzed by using two sets of phylogenetic markers, including 120 bacterial single-copy genes (Fig. 2A) [35] and the 16S rRNA gene (Fig. 2B). Phylogenetic analysis of 120 bacterial single-copy genes showed that the eight genomes fell into four anammox bacterial families, including *Ca.* Scalinduaceae (i.e. hadal_Sc_1, hadal_Sc_2, and hadal_Sc_3), *Ca.* Anammoxibacteraceae (i.e. hadal_Ab_1, hadal_Ab_2, and hadal_Ab_3), *Ca.* Subterrananammoxibiaceae (hadal_Sa_1), and *Ca.* Bathyanammoxibiaceae (hadal_Ba_1) (Fig. 2A). Anammox bacterial community in most marine environments traditionally has been suggested to mainly be affiliated with the *Ca.* Scalinduacae family. For instance, anammox bacteria in coastal sediments [59] and oxygen-deficient zones [31, 60–62] seem to be composed exclusively of *Ca.* Scalindua species within the *Ca.* Scalinduaceae family. Similarly, sedimentary anammox bacteria in the South China Sea mainly belong to *Ca.* Scalindua, although minor groups of the *Ca.* Brocadia and *Ca.* Kuenenia genera were also detected [63]. After the discovery of the *Ca.* Bathyanammoxibiaceae family [24], it became clear that its members are also prevalent in marine benthic environments, which at some locations are even more abundant than *Ca.* Scalinduaceae [64] and were also detected in Mariana Trench sediments [23]. The co-occurrence of three anammox families was also documented in one sediment core in the Atacama Trench [26]. The detection of four anammox bacterial families in hadal trench sediments clearly manifests the high diversity of anammox bacteria in this biogeochemical hotspot.

The hadal anammox bacterial genomes generally show low similarities to their close relatives from other environments. The highest AAI is 93.87% in the *Ca.* Scalinduaceae MAGs of hadal_Sc_1 and hadal_Sc_2, hadal_Ab_1 shows 88.83% AAI with hadal_Ab_2 in the *Ca.* Anammoxibacteraceae, and hadal Sa_1 exhibits a 79.12% AAI with Planctomycetes_bacterium_NC_42_111 (Table S1). These relatively low AAI (<95%) suggested potentially novel species of the *Ca.* Scalinduaceae, *Ca.* Anammoxibacteraceae, and *Ca.* Subterrananammoxibiaceae families, according to the thresholds outlined in Konstantinidis *et al*. [41]. The hadal_Sc_1, hadal_Sc_2, and hadal_Sc_3 are notably different from *Ca.* Scalindua communis based on the <95% AAI (Fig. 2A), suggesting that hadal_Sc_1, hadal_Sc_2, and hadal_Sc_3 each represents a distinct species in the *Ca.* Scalindua genera. Members of the *Ca.* Anammoxibacteraceae family exhibited pronounced genomic differences from those in the subsea tunnel biofilms (i.e. Anammoxibacter_A_OFTM134 and Anammoxibacter_B_OFTM214) [26], suggesting that hadal_Ab_1, hadal_Ab_2, and hadal_Ab_3 represented distinct species in the *Ca.* Anammoxibacteraceae family. Similarly, *Ca.* Subterrananammoxibiaceae members were distinct from those in groundwater environments based on the <95% AAI (i.e. *Ca.* Subterrananammoxibius californiae) [26]. Considering that genomes of *Ca.* Anammoxibacteraceae [25] and *Ca.* Subterrananammoxibiaceae [26] previously have only been recovered from subsea tunnel biofilm and groundwater, respectively, the recovery of hadal anammox bacterial genomes affiliated with these two families also greatly expanded their distribution ranges on Earth. Our results revealed the existence of several novel anammox bacteria in hadal trenches at the genomic level; future cultivation or single-cell genomes would be helpful to validate these speculations.

The phylogenetic analysis based on the 16S rRNA gene supports the classification of hadal anammox genomes classification, although only hadal_Sc_1, hadal_Ab_1, and hadal_Ab_2 contain the 16S rRNA gene (Fig. 2B). According to this tree, hadal_Ab_1 and hadal_Ab_2 belong to *Ca.* Anammoxibacteraceae, while hadal_Sc_1 should be a member of *Ca.* Scalinduaceae. Our findings revealed several novel lineages beyond *Ca.* Scalindua, spanning at least three additional families. This expanded the current phylogenetic framework of anammox bacteria and highlighted the hadal environment as a reservoir of previously unrecognized anammox diversity.

### Distribution and niche differentiation of four anammox bacterial families

**Figure 3:**
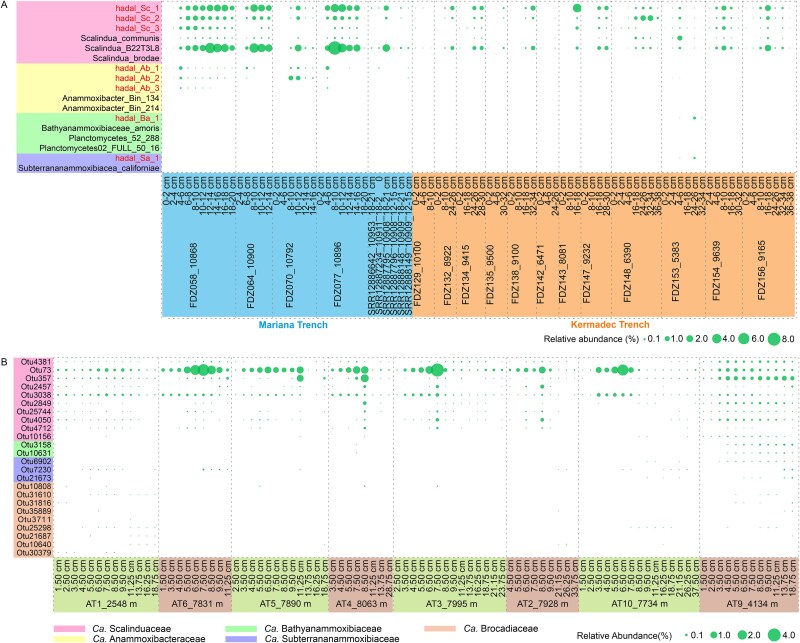
Spatial distribution of the four anammox bacterial families in sediments. MAGs in different depths of the Mariana Trench, and Kermadec Trench (A); and distribution of anammox bacteria operational taxonomic units (OTUs) in the Atacama trench (B). Bubble size corresponds to the relative abundance of each MAG, and MAGs recovered from this study are highlighted.

To explore the distribution pattern of anammox bacteria in the trenches, we used the eight genomes as the template to recruit reads from the existing metagenome sequencing data. They are more abundant in the Mariana Trench samples than in the Kermadec Trench samples (Fig. 3A), whereas they were not detected in both the Yap and the Diamantina Trenches samples. *Ca.* Scalinduaceae members, especially hadal_Sc_1, hadal_Sc_2, and *Ca.* Scalindua B22T3L8, are the most abundant ones among the eight anammox genomes and are detectable in most investigated sediment cores (Fig. 3A), indicating the dominance of this family in the anammox communities in the Mariana and Kermadec trench sediment samples. This is consistent with the assessment of Thamdrup *et al*. [7], where *Ca.* Scalindua was suggested as the dominant anammox lineage. In contrast, only trench-derived *Ca.* Anammoxibacteraceae and *Ca.* Bathyanammoxibiaceae genomes can only be occasionally detected in both Mariana and Kermadec Trench samples, and *Ca.* Subterrananammoxibiaceae seems to be uniquely present in the Kermadec Trench samples (Fig. 3A). The co-occurrence of all four families in the same core is only noticed at site FDZ153 in the Kermadec Trench samples. The low relative abundances of *Ca.* Subterrananammoxibiaceae and *Ca.* Bathyanammoxibiaceae has also been documented in Atacama Trench sediment samples [26].

To explore the distribution of anammox bacteria beyond the Mariana and Kermadec Trenches, we examined the 16S rRNA gene amplicon sequencing data derived from the Atacama Trench sediments with water depths of 2548–8063 m [30]. A total of 24 anammox bacteria Operational Taxonomic Units (OTUs) from *Ca.* Scalinduaceae*, Ca.* Subterrananammoxibiaceae, and *Ca.* Bathyanammoxibiaceae were detected. The highest proportion of OTU was ~4.86% across all 155 sediment samples. Similar to the Mariana and Kermadec Trenches, members of the *Ca.* Scalindua OTUs dominated in all the samples, especially OTU73, showing first an increase and then a decrease with sediment depths. *Ca.* Scalindua OTUs generally peaking at 6.5–8.5 cm depths before declining (Fig. 3B). *Ca.* Subterrananammoxibiaceae and *Ca.* Bathyanammoxibiaceae OTUs were predominantly recovered from 8.5 to 18.75 cm. Anammox bacteria in hadal trench sediments were dominated by *Ca.* Scalindua, while members of *Ca.* Anammoxibacteraceae and *Ca.* Subterrananammoxibiaceae were only occasionally detected in some layers with <1% relative abundances, implying that these two families had not as ecologically adapted as *Ca.* Scalindua to the hadal biosphere. We found that four out of the five known anammox bacterial families present in hadal sediments thus provided new insights into the diversity of anammox bacteria in trench sediments.

### Metabolic potentials of the four anammox families in trenches

**Figure 4:**
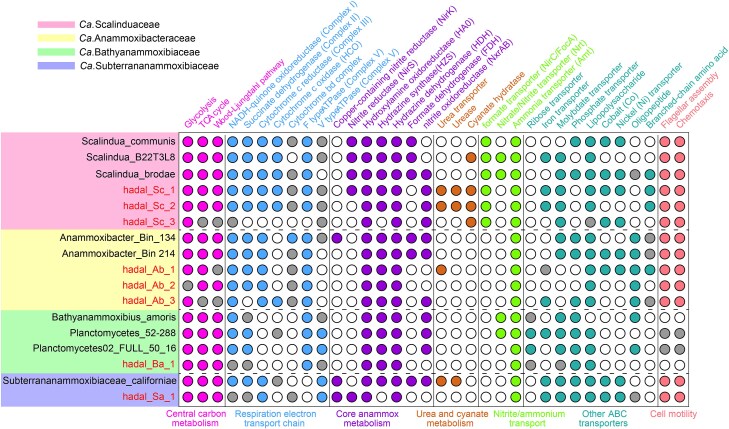
Metabolic potentials encoded in anammox bacteria genomes. Filled color circles indicate the presence of full pathways, open circles denote the absence, while light-gray circles represent the presence of partial pathways. Genomes recovered from hadal trench sediments in this study are highlighted. High-quality genomes from other environments are included for comparison. The family-level affiliations of the anammox bacteria are shown in the legend.

All members of the four anammox bacterial families contain the key genes encoding the critical enzymes for the core anammox metabolism. Notably, they possess the diagnostic hydrazine synthase (*Hzs*) [65, 66], which catalyzes the combination of ammonia and nitric oxide (NO) to produce hydrazine (Fig. 4). Multiple copies of *Hzs* genes, including the alpha, beta, and gamma subunits of HZS (*hzsA*, *hzsB*, and *hzsC*), were shown in some trenches’ anammox bacterial genomes (e.g. hadal_Sc_1, hadal_Sc_2, hadal_Ab_1, hadal_Ab_2, hadal_Ab_3, and hadal_Ba_1). Such gene duplications have also been reported in other anammox genomes, such as *Ca.* Kuenenia stuttgartiensis [67] and *Ca.* Benthoscalindua sediminis [68], with the enhanced capture ability for NH_4_^+^/NO_2_^−^ to survive in the trenches. The eight trench anammox bacteria also encode the characteristic anammox enzyme hydrazine dehydrogenase (*Hdh*), which catalyzes the oxidation of hydrazine to N_2_ [69]. In addition, *Ca.* Scalinduaceae (hadal_Sc_1, hadal_Sc_2, and hadal_Sc_3) and *Ca.* Anammoxibacteraceae (hadal_Ab_3) contained nitrite oxidoreductase (*Nxr*), which can provide electrons for carbon fixation through the Wood–Ljungdahl pathway. The absence of *Nxr* in the remaining four anammox genomes may be due to the incomplete genome nature. Branched-chain amino acids were encoded by two trench *Ca.* Scalinduaceae genomes (Fig. 4).

Notably, three trench *Ca.* Scalinduaceae genomes, e.g. hadal_Sc_1, hadal_Sc_2, and hadal_Sc_3, encode cyanate hydratase (or cyanase), an enzyme that catalyzes the decomposition of cyanate into ammonium and CO₂ [70]. The presence of cyanase in the three trench-derived *Ca.* Scalinduaceae genomes were fully assembled suggests that this metabolic trait confers the potential to degrade cyanate directly [31], and cyanate likely serves as an additional source of ammonium in trench sediments to stimulate anammox activity, like that observed in oxygen-deficient seawaters [71]. Their expression has been reported in marine microbial communities [71, 72], although not directly measured in our study. Two trench *Ca.* Scalindua genomes (e.g. hadal_Sc_1 and hadal_Sc_2) contain three subunits of urease (*UreA*, *UreB*, and *UreC*) and a urea transporter, but urease is absent from any of the *Ca.* Anammoxibacteraceae, *Ca.* Bathyanammoxibiaceae, and *Ca.* Subterrananammoxibiaceae genomes, suggesting a lineage-specific distribution of urease metabolism (Fig. 4). This finding strongly reinforces the earlier observation that urea can increase anammox rates in the oxygen-deficient zones of Eastern Tropical South Pacific [71]. This means that many trench *Ca.* Scalindua genomes may hydrolyze and assimilate urea to generate intracellular ammonium because urea serves as an essential nitrogen substrate for nitrogen-transforming microorganisms in marine ecosystems [73], primarily derived from zooplankton excretion [74] and the decomposition of cellular material [75]. Future studies through transcriptomic or proteomic analyses, enzyme activity assays, and stable isotope labeling experiments could be helpful to experimentally verify these metabolic traits.

### Compare genome analysis of the anammox bacteria

**Figure 5:**
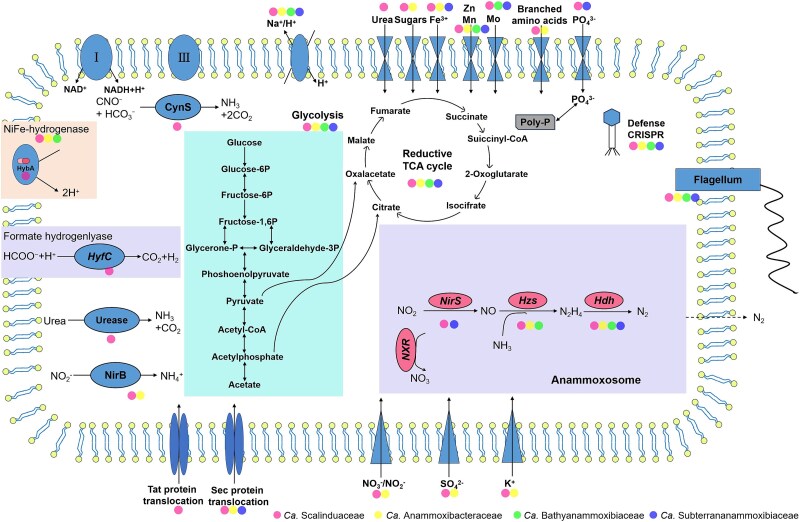
Cell metabolic cartoon constructed from the annotations of the four anammox families. Filled color indicates the presence of pathways in trench-derived anammox bacteria, including *Ca.* Scalinduaceae, *Ca.* Anammoxibacteraceae, *Ca.* Bathyanammoxibiaceae, and *Ca.* Subterrananammoxibiaceae MAGs.

Given the different distribution of inter/intra anammox bacterial families present in the trenches and non-trenches, we further compared their genomes to identify any genes that may result in this apparent population and habitat differentiation. Compared to the other five trench-derived anammox, *Ca.* Scalinduaceae genomes uniquely contain genes encoding the formate hydrogenlyase (FHL) pathway. The FHL complex was a membrane-bound multi-subunit enzyme system in facultative anaerobes, which catalyzes the disproportionation of formate into H_2_ and CO_2_ under anaerobic conditions, coupling formate oxidation with proton reduction [76]. Therefore, the detection of FHL in trench *Ca.* Scalinduaceae suggested a potential capability to utilize formate as an alternative energy source, as reported in the Mariana Trench [77] and Tonga forearc settings [78]. In addition, we found a gene cluster in trench-derived *Ca.* Scalinduaceae, compared to other environments encoding *HybA*, an Fe-S cluster-containing dehydrogenase belonging to the dimethyl sulfoxide reductase family (Fig. 5). *HybA* was likely involved in hydrogen oxidation, functioning in coordination with [NiFe] hydrogenase subunits, such as *HybB* and *HybC,* to facilitate electron transfer during anaerobic respiration as a result of adaptation to energy-limited and reductive trench environments [79, 80]. *AfuA* and *FbpB*, two components of typical ABC-type Fe^3+^ transport systems, were found to be uniquely present in the genomes of trench-derived *Ca.* Anammoxibacteraceae but absent from the genomes in the subsea tunnel biofilms (Table S2). This transport system was responsible for the high-affinity uptake of Fe^3+^ from the surrounding trench environments, and likely to maintain metabolic stability of amid fluctuations in the typical Fe solubility and redox conditions of trenches, as a possible adaptation for efficient Fe acquisition under extreme oligotrophic and redox-variable conditions [81, 82].

In addition, sulfate transporter *Cys*Z associated with sulfur metabolism was identified in the trench *Ca.* Scalinduaceae and *Ca.* Anammoxibacteraceae MAGs. This transporter is crucial for providing the sulfur source to the biosynthesis of sulfur-containing amino acids (e.g. cysteine and methionine) and the maintenance of Fe-S cluster proteins, both of which were crucial for various redox-related cellular processes. Its presence suggested that sulfate could be efficiently imported as a sulfur source for the biosynthesis of sulfur-containing amino acids and Fe-S cluster proteins, providing an advantage to *Ca.* Scalinduaceae and *Ca.* Anammoxibacteraceae in trenches, where sulfur availability and redox conditions can vary significantly [83, 84]. Furthermore, [NiFe] hydrogenases were identified in the genomes of trench *Ca.* Scalinduaceae and *Ca.* Anammoxibacteraceae, potentially enhancing the efficiency of hydrogen utilization as both an energy and electron source, indicating an enhanced capacity for hydrogen oxidation that may contribute to both energy conservation and electron transport under anoxic and low-energy conditions [80, 85]. The enrichment of ABC-type transporter systems, including those for oligopeptides and branched-chain amino acids, was detected in the trench anammox bacteria as well and very likely contributed to cellular structural stability and osmotic regulation in nutrient-limited and high-pressure environments [86].

### Divergence time of trench anammox bacteria

**Figure 6:**
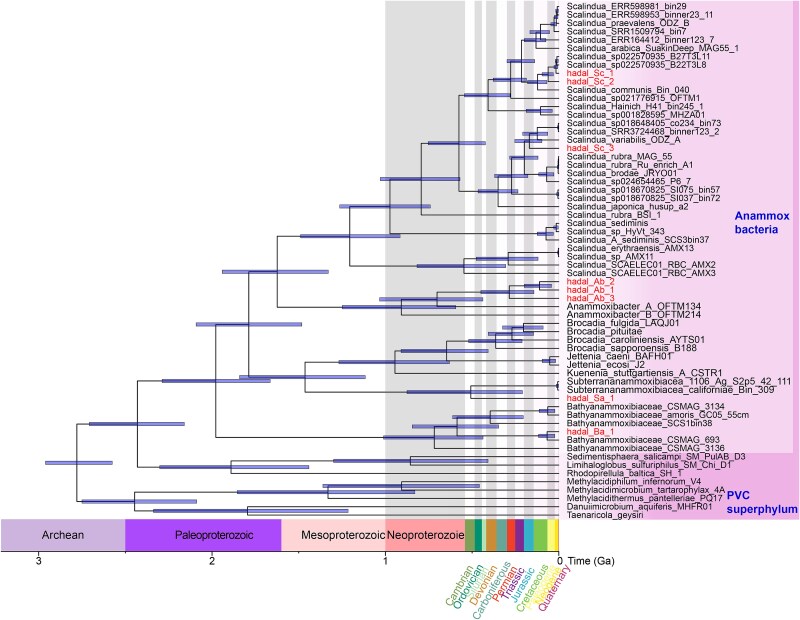
Molecular clock dating the divergence times of the four anammox families. The depicted chronogram is a time-calibrated species tree initially generated by a concatenated alignment of 26 single-copy bacterial genes. Posterior distributions were generated by sampling the key Markov Chain Monte Carlo analysis every 1000 generations, with a 25% burn-in. Horizontal bars denote 95% confidence intervals. For simplicity, only the bars for the nodes within the anammox lineage discussed in the main text are shown.

We applied MCMCTree to estimate divergence times of the four trench anammox families, yielding congruent topologies across different genome sets. Previous studies have estimated that the last common ancestor of all anammox bacteria originated between 2.32 and 2.50 billion years ago, coinciding with the Great Oxidation Event [87]. The evolutionary history of *Ca.* Scalinduaceae members in trenches showed that the origins broadly fall into the Phanerozoic Eon [95% highest posterior density (HPD) interval, 541 Ma] (Fig. 6), when oxygen levels rose during the Phanerozoic Eon, anammox became gradually confined to extreme environments such as marine sediments and oxygen-deficient zones [31]. The origins of the three trench *Ca.* Scalinduaceae bacteria (hadal_Sc_1, hadal_Sc_2, and hadal_Sc_3) were constrained in the range between 201 and 66 Ma. In particular, the origin of the crown group of the clade containing the *Ca.* Scalindua genome named hadal_Sc_3 was dated to 187 Ma (95% HPD interval, 201–173 Ma), while that of trench *Ca.* Anammoxibacteraceae clade (hadal_Ab_1, hadal_Ab_2, and hadal_Ab_3) was in the range between the Neoproterozoic and Permian (95% HPD interval, 722–290 Ma). The origin of trench *Ca.* Bathyanammoxibiaceae (hadal_Ba_1) was dated to 323 Ma (95% HPD interval, 580–66 Ma), same as *Ca.* Bathyanammoxibiaceae CSMAG 693 from clod seep [88]. Additionally, the hadal *Ca.* Subterrananammoxibiaceae species (hadal_Sa_1) were constrained in the range between Mesoproterozoic and Cambrian (95% HPD interval, 1460–513 Ma), which originated earlier than the other two MAGs from groundwater (Fig. 6).

The divergence time of trench anammox bacteria was prior to the formation of hadal trenches. For example, the ages of the Mariana Trench and Kermadec Trench were estimated to be formed ~55 Ma [89] and ~25 Ma [90], respectively, while anammox bacteria in trencher were estimated to be 1460–66 Ma. Considering that the deep ocean has been oxygenated since ~420 Ma [91], it is likely that anammox bacteria in the trench sediments were not originally from the trench but were transported from somewhere else. As the two proximities of hadal trenches, seawater and shallower sediments could be the best candidates of the sources of anammox bacteria. Seawater can circulate and disperse organisms into the hadal realm and the underlying sediments, while shallower sediments can be brought into hadal trenches by earthquakes and mass wasting [92, 93]. Since anammox bacteria in seawater prefer a free-living lifestyle [94, 95] and are confined within the oxygen-deficient zones [31], it is not highly likely that they could have existed in the overlying water column of the four trenches and get deposited onto the trench bottom due to sedimentation because none of the four investigated areas harbor an oxygen-deficient zone. Therefore, the episodic wasting of shallower sediment is the more likely origin of the anammox bacteria found in hadal sediments. Geological and sedimentological evidence indicates that hadal trenches, including the Mariana and Kermadec Trenches, function primarily as depositional centers rather than as evolutionary cradles. Steep slopes, V-shaped topography, and subduction-zone dynamics facilitate frequent mass-wasting events, which transport sediments and associated microorganisms from shallower sediments or adjacent slopes into the trench axis [96–99].

## Conclusion

Here, we reconstructed eight high-quality anammox bacteria MAGs from the sediments’ metagenomic data of the Diamantina, Kermadec, Mariana, and Yap Trenches. We showed that the eight anammox bacteria fell within four families, and seven of them were novel members. In addition to their high diversity in trench sediments, the dominant *Ca.* Scalinduaceae are phylogenetically close to those found in shallow coastal sediments and oxygen-deficient zones. Comparative genomic analysis showed that trench-derived *Ca.* Scalinduaceae genomes could utilize cyanate, urea, and formate besides ammonium, and contained the genes for hydrogen oxidation via [NiFe] hydrogenase, supporting them to thrive in the hadal environment. The evolutionary radiation of these anammox bacteria was estimated to have happened <1.46 Ga. The first anammox bacteria in hadal sediments probably derived from shallower sediments and were transported into the trenches by sediment wasting. This study provided new insights into the unique features of the ecology and history of the microbial nitrogen cycling processes in the deepest part of the ocean. Further laboratory incubation experiments and *in situ* activity tests would be needed to verify the contribution of hadal microbial communities to the global nitrogen biogeochemical cycles.

## Ethics and consent for publication

All sediment samples analyzed in this study were collected during legally authorized scientific expeditions. Samples from the Kermadec and Diamantina Trenches were obtained under the Global Trench Exploration and Diving Programme (Global TREnD). All sampling activities were conducted in compliance with international and national regulations governing marine scientific research within the respective Exclusive Economic Zones (EEZs), with permissions issued by New Zealand.

## Supplementary Material

Supplementary_Table_ycag011

## Data Availability

The raw metagenomic sequencing reads have been deposited in the NCBI Sequence Read Archive (SRA) under BioProject accession number PRJNA1187693 and PRJNA1111327. The eight trench anammox genomes are available in the NCBI database under project ID PRJNA1291704, with genome accession numbers of JBRBQJ000000000 (hadal_Sc_1), JBRBQK000000000 (hadal_Sc_2), JBRBQL000000000 (hadal_Sc_3), JBRBQM000000000 (hadal_Ab_1), JBRBQN000000000 (hadal_Ab_2), JBRBQO000000000 (hadal_Ab_3), JBRBQP000000000 (hadal_Ba_1), and JBRBQQ000000000 (hadal_Sa_1).
